# Application of a Navigated Drill for Cervical Pedicle Screw Insertion at C3-6

**DOI:** 10.7759/cureus.47710

**Published:** 2023-10-26

**Authors:** Tomoyuki Takigawa, Takuya Morita, Yuya Kajiki, Yasuo Ito

**Affiliations:** 1 Department of Orthopaedic Surgery, Kobe Red Cross Hospital, Kobe, JPN

**Keywords:** vertebral artery injury, navigated probe, navigated drill, perforation rate, cervical pedicle screw

## Abstract

Background

Perforation of the cervical pedicle screw, especially lateral perforation, may lead to critical complications, such as vertebral artery injury. Sub-axial cervical spines (C3-6) are at risk of complications because these levels have limited area and angle. This study aimed to compare a navigated drill and a navigated probe for the insertion of cervical pedicle screws at C3-6.

Methodology

This retrospective study included 106 patients treated with cervical pedicle screws at C3-6. In total, 52 patients with 200 cervical pedicle screws using a navigated drill (group D) and 54 patients with 170 cervical pedicle screws using a navigated probe (group P) were compared. The perforation rate, anatomical medial angle of the pedicle, and actual angle of the screw were investigated using computed tomography. The planning error was defined as when the pedicle screw was applied for a small pedicle width of <3.5 mm. All perforations except for planning errors were defined as technical perforations.

Results

Grade 1 screw perforations were identified in 16 and 17 screws in groups D and P, respectively. Overall, 88% of the perforations were medial in group D, and 82% of perforations were lateral in group P. Technical perforations were found in 7/191 (3.7%, group D) and 15/168 (8.9%, group P) screws. There were no significant differences in the anatomical angle of the pedicle between the groups. The mean medial angle of the pedicle screws was 24.7° (group D) and 30.9° (group P) (p < 0.05).

Conclusions

The perforation rate of group D was less than half of that of group P. This was because a navigated drill was able to create a bony pilot hole at the hard medial cortical wall of the pedicle with a small medial angle, which was difficult to do with a navigated probe. A navigated drill can be useful for cervical pedicle screw insertion at C3-6 because of its easiness and safety.

## Introduction

Cervical pedicle screw fixation is gaining popularity because it is the most biomechanically rigid method for cervical fixation [1]. In addition to meticulous preoperative planning to identify the ideal entry point and trajectory, thorough understanding and expertise are required to succeed in cervical pedicle screw insertion [2]. The main concern with the use of cervical pedicle screws is the associated complications. Perforation by the cervical pedicle screw poses the risk of neurological or vascular injury. In particular, lateral perforation may lead to vertebral artery injury, which may cause cerebellar infarction and even death. The sub-axial cervical spine (C3-6) is prone to complications because these levels provide a limited area and angle. The use of a navigation system has been shown to improve accuracy [3], but not sufficiently. There are many processes involved in navigation. The most important of these is creating a pilot hole in the vertebral pedicle, which is generally done using a navigated probe. A navigated drill was released in 2016, and its utilization in creating a pilot hole has been shown to decrease lateral perforation compared to a navigated probe [4]. However, there is no report on a large number of cases involving navigated drills. We hypothesized that a navigated drill would improve safety by minimizing the perforation rate. This study aimed to investigate and compare a navigated drill with a navigated probe for the insertion of cervical pedicle screws at C3-6.

This article was previously presented as a meeting abstract at the 50th annual meeting of the Japanese Society for Spine Surgery and Related Research on April 22-24, 2021, the 94th annual meeting of the Japanese Orthopaedic Association on May 20-23, 2021, AO SPINE Japan Conference/Congress on August 28, 2021, and the 12th annual meeting of Cervical Spine Research Society - Asia Pacific on June 24-25, 2022.

## Materials and methods

**Figure 1:**
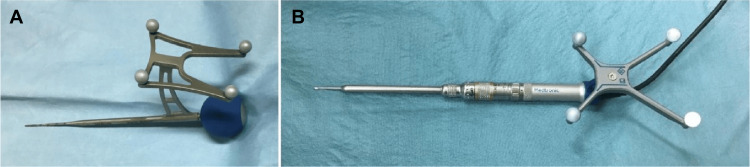
(A) Navigated probe. (B) Navigated drill.

A total of 106 consecutive patients treated at our institute with cervical pedicle screws at C3-6 were retrospectively investigated. All cases in which at least one cervical pedicle screw was inserted from C3 to C6 were included in this study. A navigated probe (Medtronic, Minneapolis, MN) was used from 2016 to 2018 as a preparation tool for cervical pedicle screw insertion, whereas a Stealth-Midas navigated drill (Medtronic, Minneapolis, MN) was used from 2018 to 2021 (Figure 1).

**Figure 2:**
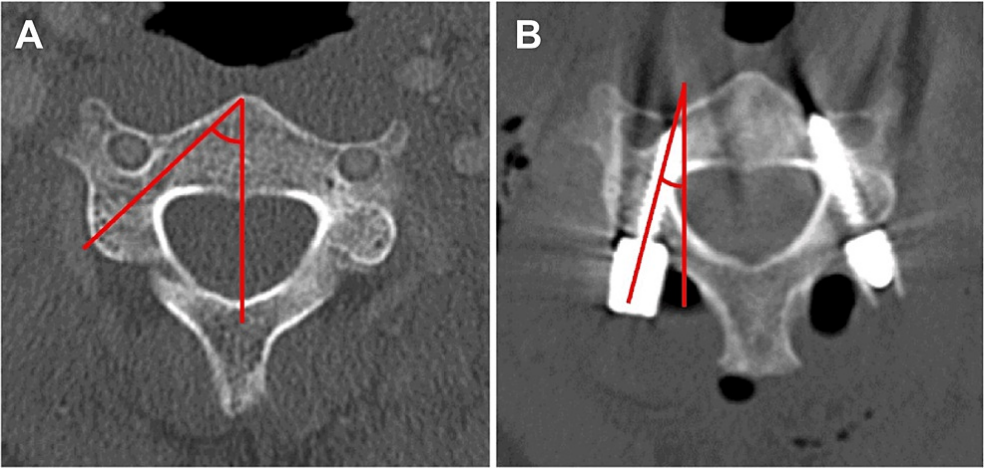
Preoperative and postoperative computed tomography (CT). The anatomical medial angle of the pedicle (A) was measured using preoperative CT. The actual angle of the screw (B) was measured using postoperative CT.

Patient background, etiology for cervical spine surgery, and accuracy of cervical pedicle screws were investigated. We compared 52 patients with 200 cervical pedicle screws using a navigated drill (group D) and 54 patients with 170 cervical pedicle screws using a navigated probe (group P). The perforation rate (Neo classification [5], grade 0: none, grade 1: <2 mm, grade 2: 2-4 mm, grade 3, >4 mm), anatomical medial angle of the pedicle, and actual angle of the screw were investigated using preoperative and postoperative computed tomography (CT) (Figure 2).

The minimal screw size was 3.5 mm and its perforation was unavoidable when the pedicle diameter was less than 3.5 mm. Thus, planning error was defined as when the pedicle screw was applied for a small pedicle width of <3.5 mm. All perforations except for planning errors were defined as technical perforations. The technical perforation rate was the primary outcome of this study. In addition to postoperative CT data, clinical and operative records of the screw perforations with their directions were investigated. The Ethics Committee of Kobe Red Cross Hospital approved this study (approval number: 2023-316). All patient information was anonymized, and informed consent was obtained from all patients.

Surgical methods

**Figure 3:**
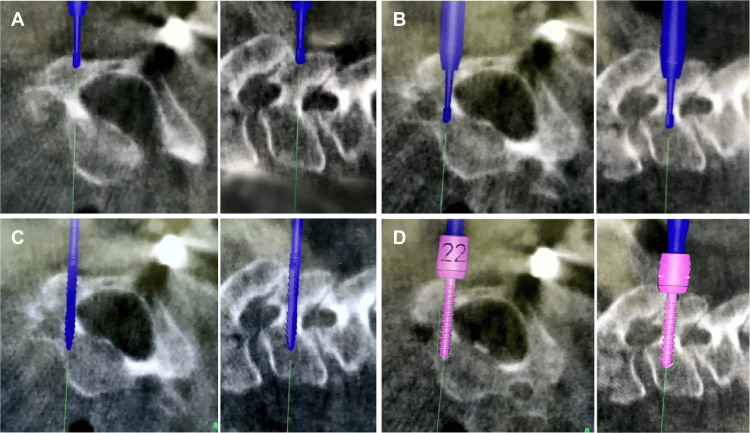
Cervical pedicle screw insertion using a navigated drill. A navigated drill was used to make a starting hole (A) and a pilot hole through the pedicle to the vertebral body (B). A navigated tap was used to tap and measure the actual screw length (C). A pedicle screw was inserted using a navigated screwdriver (D). The left side shows the axial image and the right side shows the sagittal image in each panel (A-D).

A radiolucent skull clamp was used to stabilize the skull, and the cervical alignment was checked before surgery. After a general midline incision and exposure of the target vertebrae, the reference frame was attached to the spinous process of this level. When the spinous process was fractured or unstable, an adjacent level was used for the reference frame attachment. Arcadis Orbic 3D (Siemens, Munich, Germany) was used to acquire CT data, and Stealth Station S7 (Medtronic, Minneapolis, MN) was used to reconstruct the data for navigation. Multiple CT scans of each vertebra were performed when the cervical spine was unstable. For example, in the case of C4/5 dislocations, the pedicle screws at C4 were inserted using navigation data acquired with the reference frame attached to C4, and the pedicle screws at C5 were inserted using other navigation data acquired with the reference frame attached to C5. During the preparation and insertion of the pedicle screw, care was taken not to penetrate the cortical wall of the pedicle, especially the external cortex, in both groups. In group P, the starting point was determined using a navigated pointer and prepared using a high-speed burr. A navigated probe was then used to create a pilot hole from the starting point through the pedicle to the vertebral body. In group D, a navigated drill was used to create a starting hole and a pilot hole (Figure 3).

The drill tip of the navigated drill was steel and 2.2 mm in diameter. The rotational frequency of the navigated drill was set at 10,000 rpm. A pilot hole was drilled through the pedicle from the starting hole to the vertebral body. In both groups, a pedicle screw was inserted using a navigated screwdriver (Medtronic, Minneapolis, MN) after tapping with a navigated tap (Medtronic, Minneapolis, MN). All procedures were performed by an attending board-certified spinal surgeon. While the first choice for an anchor was the cervical pedicle screw, the decision was made by the surgeon regarding whether to apply the cervical pedicle screws and concerning the thickness and length.

Statistical analysis

The perforation rate and direction were analyzed using the chi-square test. Patient background data (age, sex ratio, and diagnosis) and the angles of the screw and pedicle were analyzed using the t-test. Parametric tests were performed based on normally distributed data. Statistical differences were considered significant when the p-value was <0.05. Excel 2016 (Microsoft, Redmond, WA) was used for the statistical analysis.

## Results

Patient backgrounds are listed in Table 1. The mean age was 60.7 years in group D and 64.3 years in group P, with no significant differences. There was a male predominance in both groups (77% in group D and 74% in group P). Cervical trauma was the main reason for the surgery in both groups (87% in group D and 89% in group P), followed by cervical spinal myelopathy, cervical spinal metastasis, and ossification of the posterior longitudinal ligament.

**Table 1:** Demographic data of patients. CSM = cervical spinal myelopathy; OPLL = ossification of the posterior longitudinal ligament

	Number of cases	Mean age (years)	Male ratio	Diagnosis
Group D	52	60.7	77%	Trauma 45, Metastasis 4, CSM 2, OPLL 1
Group P	54	64.3	74%	Trauma 48, CSM 6

The accuracy of the cervical pedicle screws is presented in Table 2 and Table 3. No grade 2 or 3 perforations were found in any of the groups. Grade 1 screw perforations were identified in 16 screws in group D and 17 screws in group P. No clinical complications related to screw malpositioning were observed; 88% of perforations were medial in group D and 82% were lateral in group P (p < 0.01). No superior or inferior perforation was identified in any of the groups. Planning error was found in nine pedicles in group D and two pedicles in group P, which was not significantly different (p = 0.06). Technical perforation was found in seven of 191 screws (3.7%) in group D and 15 of 168 screws (8.9%) in group P, with a significant difference (p = 0.038). The mean anatomical angle of the pedicle was 46.3° in group D and 46.4° in group P, which was not significantly different (p = 0.89). The mean angle of the pedicle screws was 24.7° in group D and 30.9° in group P, which was significantly different (p < 0.01).

**Table 2:** Accuracy of cervical pedicle screws. *: Technical perforation indicates perforation when the pedicle width is more than 3.5 mm. Detail is explained in the main text. †: p < 0.01; ‡: p < 0.05.

	Grades 2–3 (> 2 mm)	Grade 1 (<2 mm)	Medial perforation	Lateral perforation	Technical perforation*
Group D	0% (0/200)	8% (16/200)	88% (14/16)†	12% (2/16)	3.7% (7/191)‡
Group P	0% (0/170)	10% (17/170)	18% (3/17)	82% (14/17)	8.9% (15/168)

**Table 3:** Technical perforation of cervical pedicle screws. The values are represented as perforation/total number of screws on each side of the cervical level.

	C3		C4		C5		C6		Total
	Right	Left	Right	Left	Right	Left	Right	Left	
Group D	0/17	0/15	0/19	0/18	1/37	3/32	2/27	1/26	7/191
Group P	2/10	1/10	4/19	1/21	3/27	2/24	1/30	1/27	15/168

## Discussion

The technical perforation rate in group D was 3.7% and was significantly smaller than that in group P (8.9%). No grade 2 or 3 perforations were found in any group. The mean medial angle of the pedicle screws in group D was 24.7°, which was significantly smaller than that in group P (30.9°). There are similar reports of increased accuracy with the use of navigated drills. Satake et al. investigated 207 cervical pedicle screws in 47 cases and showed the usefulness of a navigated drill for cervical pedicle screw placement with reduced perforation in the lateral and rostral directions [4]. Our results, with a perforation rate of 3.7%, were superior to the results of Satake et al. with a perforation rate of 7.2% in the axial plane and 10.5% in the sagittal plane. We attribute this to the fact that most of our cases were traumatic, less degenerative, and involved younger people.

Abumi et al. first reported a clinical series of cervical pedicle screw applications, with a perforation rate of 6.7% using lateral fluoroscopy [6]. A wide range of screw perforation rates, depending on screw insertion methods and guidance systems, has been reported. Neo et al. recommended the refinement of screw insertion, such as a navigation system with a high perforation rate of 29% [5]. Ito et al. utilized a three-dimensional fluoroscopy-assisted navigation system and reported a low perforation rate of 2.8% [3]. Yukawa et al. developed the pedicle axis view and reported a 14% perforation rate [7]. Chachan et al. utilized an O-arm-based navigation system and reported high accuracy, with a 7.0% perforation rate [8]. Most previously reported studies on cervical pedicle screw accuracy include C2 and C7, which are relatively wider and easier than C3-6. Our results, with a technical perforation rate of 3.7%, are favorable because our data included only C3 to C6 and excluded C2 and C7.

Medial perforations are reportedly safer than lateral perforations. This is because the medial distance from the pedicle to the spinal cord is 6.56 mm and wider than the lateral distance from the pedicle to the vertebral artery which is 0.89 mm [9]. This is well-known to spinal surgeons. However, lateral perforations are more common in the clinical setting, considering our results in group P. Several reasons have been proffered. First, the medial cortical wall of the cervical pedicle is 1.4 to 3.6 times thicker than the lateral wall of the pedicle [10]. Second, retracted muscles resist the insertion of the pedicle screw from the angle required by the surgeon [5]. Lastly, insertion torque has the risk of rotating the vertebra, which results in lateral perforation [11].

Pedicle probes are widely used to create pilot holes in the pedicles. Pedicle probes are designed to transmit a straight force and tunnel through the cancellous bone of the pedicle. This concept applies to the thoracic and lumbar pedicles, but not to the cervical pedicles, as mentioned above. The trajectory of the cervical probe tends to change in the lateral direction. Abumi et al. described an increase in the acceptable range of angles by drilling and digging a lateral mass [12]. Mahesh et al. reported the importance of drilling the medial part of the pedicle [13]. Navigated drilling enables the creation of a pilot hole at the medial edge of the pedicle, which is difficult even with a navigated probe. Furthermore, the navigated drill method does not require excessive power or exposure to the lateral part of the cervical spine. This unique method resulted in a low perforation rate in the lateral direction and an approximately half angle of the screw trajectory compared with the anatomical pedicle angle.

We believe that the key point of this navigated drill method is to maintain the straightness of the drill tip at a relatively small revolution speed. We usually use a 2.2-mm-sized drill tip and a rotational frequency of 10,000 rpm. Minimizing out-of-orbit vibration and oscillation of the drill tip ensured navigation accuracy. Rough and forceful penetration at a higher rotation speed tends to result in inaccuracies and dangers.

The limitations of this study include the small number of cases, the single-institute design, and the retrospective nature. We did not have a perfect match for the surgeon, patient background, anatomy, etiology, planning errors, and other factors between the two groups, which might have influenced the results. A prospective, large-scale, multicenter study is required before the wide acceptance of this study.

## Conclusions

A navigated drill could minimize the perforation rate compared with a navigated probe. This was because a navigated drill was able to create a bony pilot hole at the hard medial cortical wall of the pedicle with a small medial angle, which was difficult to do with a navigated probe. A navigated drill can be useful for cervical pedicle screw insertion at C3-6 because of its ease and safety.
